# Marital status and post-radical prostatectomy outcomes: results from the SEARCH database

**DOI:** 10.1007/s00520-026-10783-y

**Published:** 2026-05-19

**Authors:** Alanna Burwell, Jaruda Ithisuphalap, Jessica L. Janes, Vanessa M. Helms, Christopher L. Amling, William J. Aronson, Matthew R. Cooperberg, Christopher J. Kane, Zachary Klaassen, Martha K. Terris, Lourdes Guerrios Rivera, Stephen J. Freedland, Alix G. Sleight

**Affiliations:** 1 Section of Urology, Durham VA Health Care System, Durham, NC USA; 2 Department of Urology, Oregon Health and Science University, Portland, OR USA; 3 Section of Urology, West Los Angeles VA Medical Center, Los Angeles, CA USA; 4 Section of Urology, San Francisco VA Medical Center, San Francisco, CA USA; 5 Department of Urology, Cedars-Sinai Medical Center, Los Angeles, CA USA; 6 Department of Urology, University of California, San Diego, CA USA; 7 Division of Urology, Department of Surgery, Augusta University-Medical College of Georgia, Augusta, GA USA; 8 Section of Urology, Charlie Norwood VA Medical Center, Augusta, GA USA; 9 Section of Surgery, VA Caribbean Healthcare System, San Juan, PR USA; 10 Division of Occupational Science and Occupational Therapy, University of Southern California, Los Angeles, CA USA

**Keywords:** Prostate cancer, Social support, Marital status, Prostate cancer outcomes, Veterans

## Abstract

**Purpose:**

Social support, specifically marital status, has been shown as a significant prognostic factor for survival of multiple malignancies, including prostate cancer. However, this has not been investigated in an equal access Veterans Affairs (VA) cohort where other support systems exist that may minimize the potential benefit of social support from a partner.

**Methods:**

We retrospectively reviewed data from 9,931 patients undergoing primary radical prostatectomy (RP) in the VA from 1988–2020 across 9 VA centers. Univariable and multivariable Cox proportional hazards models were used to test the association between marital status and biochemical recurrence (BCR), metastasis, castration-resistant PC (CRPC) and prostate cancer specific mortality (PCSM).

**Results:**

8,285 patients met the inclusion criteria: 54% were married, 30% were divorced/separated, 9% were single/never married, and 6% were widowed at the time of RP. Single/never married men were younger (median 61 vs 62–65 years), had surgery more recently (median 2009 vs 2003–2008), had higher PSA (median 6.9 ng/mL vs 6.4–6.8 ng/mL), and had lower BMI (median 27 vs 28) compared to other groups (all p < 0.05). The median time to BCR was significantly shorter for divorced/separated men (188.2 months) and single/never married men (154.8 months) compared to married men (243.0 months). Consistent with this finding, compared to married men, divorced/separated men had higher risk of BCR (HR = 1.12; 95% CI 1.03–1.21), as did single/never married men (HR = 1.13; 95% CI 1.00–1.28). However, these associations were insignificant in multivariable analyses (all p > 0.05).

**Conclusion:**

Among men with localized prostate cancer undergoing RP within the VA, we found no association between marital status—defined as a demographic indicator of self-reported relationship category—and oncologic outcomes. Whether marital satisfaction or perceived partner support, which were not assessed in this study, influence post-RP outcomes remains to be investigated.

## Introduction

Prostate cancer ranks number one for estimated new cases of cancer for men and ranks second for estimated cause of death[1]. Treatment is based on life expectancy and risk of death from comorbidities. Primary treatment options for patients with localized disease include surveillance and potentially curable therapies such as surgery and radiation. Surgery such as radical prostatectomy (RP) is a standard option which provides tumor control for many patients[2]. The side effects from RP include erectile dysfunction and urinary incontinence[3], thus making the need for social support crucial to help individuals cope.

Social support, particularly from family members, can improve cancer outcomes and quality of life[4, 5]. A cancer diagnosis is often challenging and life-changing; it can disrupt daily activities and long-term plans while bringing about financial burdens, leading to depression and anxiety for the patients and their family and friends[6]. Specifically, a diagnosis of prostate cancer diagnosis is linked to feelings of depression and anxiety and increased risk of suicide[3, 7, 8]. Emotional support plays a critical role in helping individuals adjust psychologically to cancer and choosing the most suitable treatment option.[9]. Research indicates that unmarried cancer patients are more likely to be diagnosed with an advanced-stage disease compared to married patients and less likely to receive aggressive treatment[10–12]. Advanced-stage diagnoses can be due to a lack of support and encouragement to go for early check-ups or screenings that lead to early diagnosis.[13]. In addition, married patients often have a higher socio-economic status compared to non-married patients, enabling them to have better insurance and better access to healthcare[14], which can lead to better cancer outcomes. For non-married individuals or those who do not have social support, these outcomes can be similar to those of married individuals, if not better, with information support and economic and social policies put in place[15]. Having support, such as spousal, can help with transportation, managing paperwork, handling household chores, and sharing the financial load.

Many studies have researched the association of marital status as a prognostic factor for the survival of multiple malignancies, including prostate cancer [12, 16–21]. However, this has not yet been investigated in a Veterans Affairs (VA) cohort where other support systems offered by the Veterans Affairs Health Care System (VAHCS) may equalize the potential social support provided by a partner. This study tested whether there is an association between marital status and RP outcomes in an equal-access VA cohort.

## Methods

### Data source and patient population

**Fig. 1:**
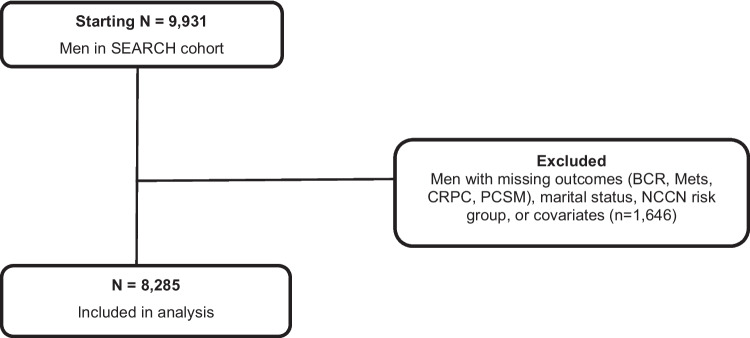
Initial cohort and exclusion criteria

After obtaining IRB approval with waiver of consent from the Durham VA IRB, we conducted a retrospective analysis of patients who underwent RP between 1988 and 2020 using the Shared Equal Access Regional Cancer Hospital (SEARCH) database. The SEARCH cohort is a racially diverse cohort with prostate cancer who were treated with RP at nine Veterans Affairs hospitals nationwide[22]. For this study, we excluded patients with missing outcomes of interest, or covariates entered into the multivariable models (n = 1,646) (Fig. 1). This resulted in an analysis cohort of 8,285 patients (54% were married, 30% were divorced/separated, 9% were single/never married, and 6% were widowed at the time of RP). We used NCCN guidelines version 4.2022 for prostate cancer to categorize patients into low- to high-risk prostate cancer[23]. For staging, patient data were reclassified, and for grade, we used the clinical diagnostic grade reported by the standard-of-care pathology at the time of surgery. No effort was made to re-review or regrade the tumor. Marital status was defined by self-reported relationship category at the time of RP (married, divorced/separated, single/never married, or widowed). This variable captures structural relationship status only and does not reflect relationship quality, perceived partner support, or marital satisfaction, which were not assessed in this study.

### Statistical analysis

The distributions of demographic and clinical characteristics were compared across four marital status groups (married, divorced/separated, single/never married, and widowed) using Kruskal–Wallis tests for continuous variables and chi-squared tests for categorical variables. The Kaplan–Meier method was used to estimate survival probabilities over time with time of surgery as time zero. The association between marital status and risk of biochemical recurrence (BCR), metastasis, castration resistant PC (CRPC), and PC specific mortality (PCSM) were evaluated using univariable and multivariable Cox proportional hazards models. BCR was defined as a single PSA > 0.2 ng/mL, two concentrations at 0.2 ng/mL, or salvage treatment for an elevated postoperative PSA. Metastases were identified typically by bone scan or computer tomography (CT) imaging performed as per the discretion of the treating clinician and assessed by trained personnel to determine the development of metastases. CRPC was defined as an elevated PSA of ≥ 2 ng/mL and ≥ 25% from the post-androgen deprivation therapy PSA nadir while being castrate, defined as serum testosterone < 50 ng/dL, bilateral orchiectomy, or continuous receipt of luteinizing hormone releasing hormone agonist or antagonist (CITE). PCSM was defined as death with metastatic progressive CRPC, without another obvious cause of death. For PCSM, competing risk models were used to account for death from other causes.

Two multivariable cox proportional hazards models were tested in this study. The first model was adjusted for clinical characteristics such as age at time of surgery (continuous, years), race (Black, White, or Other), year of surgery (continuous), biopsy Gleason score (2–6, 7, or 8–10), clinical stage (T1, T2, or T3/T4), and pre-operative PSA (continuous, ng/mL, log-transformed). The second model was adjusted for pathological features such as RP Gleason score (2–6, 7, or 8–10), pre-operative PSA (continuous, ng/mL, log-transformed), seminal vesicle invasion, positive surgical margin, lymph node involvement (positive, negative, or not performed), extracapsular extension, and pathological stage (T0-T2, T3, or T4). Kaplan–Meier plots were constructed to compare BCR, Mets, CRPC across marital status groups. Cumulative incidence curve was constructed to compare PCSM across marital status groups. K-sample tests were used to assess differences in cumulative incidence.

In a sensitivity analysis, the analysis described above was repeated after additional adjustment for the time from diagnosis to surgery among a cohort of men (N = 8,215) who had diagnosis data available. All multivariable models remained the same with the addition of the log-transformed number of days from diagnosis to RP.

All statistical tests were 2-sided, and all analyses were performed using SAS Enterprise Guide version 8.3 software (SAS Institute, Cary, NC).

## Results

Baseline demographics and clinical characteristics of patients in the four marital status groups can be found in Table 1. Single/never married men were younger (median 61 vs 62–65 years), had surgery more recently (median 2009 vs 2003–2008), had higher PSA (median 6.9 ng/mL vs 6.4–6.8 ng/mL), and had lower BMI (median 27 vs 28) compared to other groups (all p < 0.05). Widowers were more likely to have stage cT2-T4, less likely to have pre-op Gleason score 7–10, and had the shortest time from diagnosis to RP (median = 84 vs. 95–101 days) compared to other groups (all p < 0.05).

**Table 1:** Patient characteristics by marital status

	Married (N = 4,499)	Divorced/Separated (N = 2,510)	Single/Never Married (N = 768)	Widowed (N = 508)	p-value
**Age**, years					< 0.001^a^
Median (IQR)	63 (59, 67)	62 (57, 66)	61 (56, 65)	65 (61, 69)	
**Race**, n (%)					< 0.001^b^
White	3117 (69%)	1640 (65%)	448 (58%)	340 (67%)	
Black	1212 (27%)	809 (32%)	300 (39%)	155 (31%)	
Other	170 (4%)	61 (2%)	20 (3%)	13 (3%)	
**Year of surgery**					< 0.001^a^
Median (IQR)	2009 (2003, 2014)	2008 (2003, 2013)	2009 (2003, 2013)	2003 (1998, 2009)	
**PSA, (ng/mL)**					< 0.001^a^
Median (IQR)	6.4 (4.8, 9.5)	6.8 (4.9, 10.3)	6.9 (4.9, 10.7)	6.7 (4.9, 10.9)	
**Clinical stage**, n (%)					0.005^b^
T1	2507 (56%)	1372 (55%)	430 (56%)	244 (48%)	
T2	1950 (43%)	1118 (45%)	332 (43%)	253 (50%)	
T3/T4	42 (1%)	20 (1%)	6 (1%)	11 (2%)	
**Pre-op gleason score**, n (%)					< 0.001^b^
2–6	1777 (39%)	958 (38%)	297 (39%)	267 (53%)	
7	1986 (44%)	1098 (44%)	343 (45%)	173 (34%)	
8–10	736 (16%)	454 (18%)	128 (17%)	68 (13%)	
**BMI**					< 0.001^a^
Median (IQR)	28 (26, 31)	28 (25, 31)	27 (24, 31)	28 (25, 31)	
**NCCN risk group**, n (%)					< 0.001^b^
Very Low	250 (6%)	127 (5%)	52 (7%)	39 (8%)	
Low	1067 (24%)	558 (22%)	158 (21%)	138 (27%)	
Favorable Intermediate	774 (17%)	386 (15%)	122 (16%)	70 (14%)	
Unfavorable Intermediate	1250 (28%)	733 (29%)	243 (32%)	118 (23%)	
Unknown Intermediate	221 (5%)	115 (5%)	27 (4%)	38 (7%)	
High	798 (18%)	510 (20%)	142 (18%)	91 (18%)	
Very High	139 (3%)	81 (3%)	24 (3%)	14 (3%)	
**Post-op gleason score**, n (%)					< 0.001^b^
2–6	1144 (25%)	658 (26%)	203 (26%)	182 (36%)	
7	2693 (60%)	1487 (59%)	446 (58%)	256 (50%)	
8–10	662 (15%)	365 (15%)	119 (15%)	70 (14%)	
**Seminal vesicle invasion**, n (%)					0.007^b^
No	4032 (90%)	2231 (89%)	657 (86%)	459 (90%)	
Yes	467 (10%)	279 (11%)	111 (14%)	49 (10%)	
**Positive surgical margin**, n (%)					0.699^b^
No	2894 (64%)	1596 (64%)	478 (62%)	322 (63%)	
Yes	1605 (36%)	914 (36%)	290 (38%)	186 (37%)	
**Lymph node involvement**, n (%)					0.055^b^
No	3004 (67%)	1727 (69%)	531 (69%)	346 (68%)	
Yes	139 (3%)	95 (4%)	26 (3%)	9 (2%)	
Not done	1356 (30%)	688 (27%)	211 (27%)	153 (30%)	
**Extracapsular extension**, n (%)					0.779^b^
No	3419 (76%)	1889 (75%)	591 (77%)	383 (75%)	
Yes	1080 (24%)	621 (25%)	177 (23%)	125 (25%)	
**Pathological stage**, n (%)					0.833^b^
T0-T2	3135 (70%)	1740 (69%)	524 (68%)	344 (68%)	
T3	1097 (24%)	622 (25%)	189 (25%)	133 (26%)	
T4	267 (6%)	148 (6%)	55 (7%)	31 (6%)	
**Days from diagnosis to RP**					< 0.001^b^
Median (IQR)	95 (64, 141)	101 (69, 150)	98 (68, 152)	84 (55, 138)	

^a^Kruskal-Wallis p-value

^b^Chi-Square p-value

The median duration of follow-up was significantly longer for widowers (154 months) compared to other groups (108 months for married men, 112 months for divorced/separated men, and 114 months for single/never married men; p < 0.001). During this time, 1,609 (35.8%) married men, 973 (38.8%) divorced/separated men, 308 (40.1%) single/never married men, and 193 (38.0%) widowed men developed BCR.

**Fig. 2:**
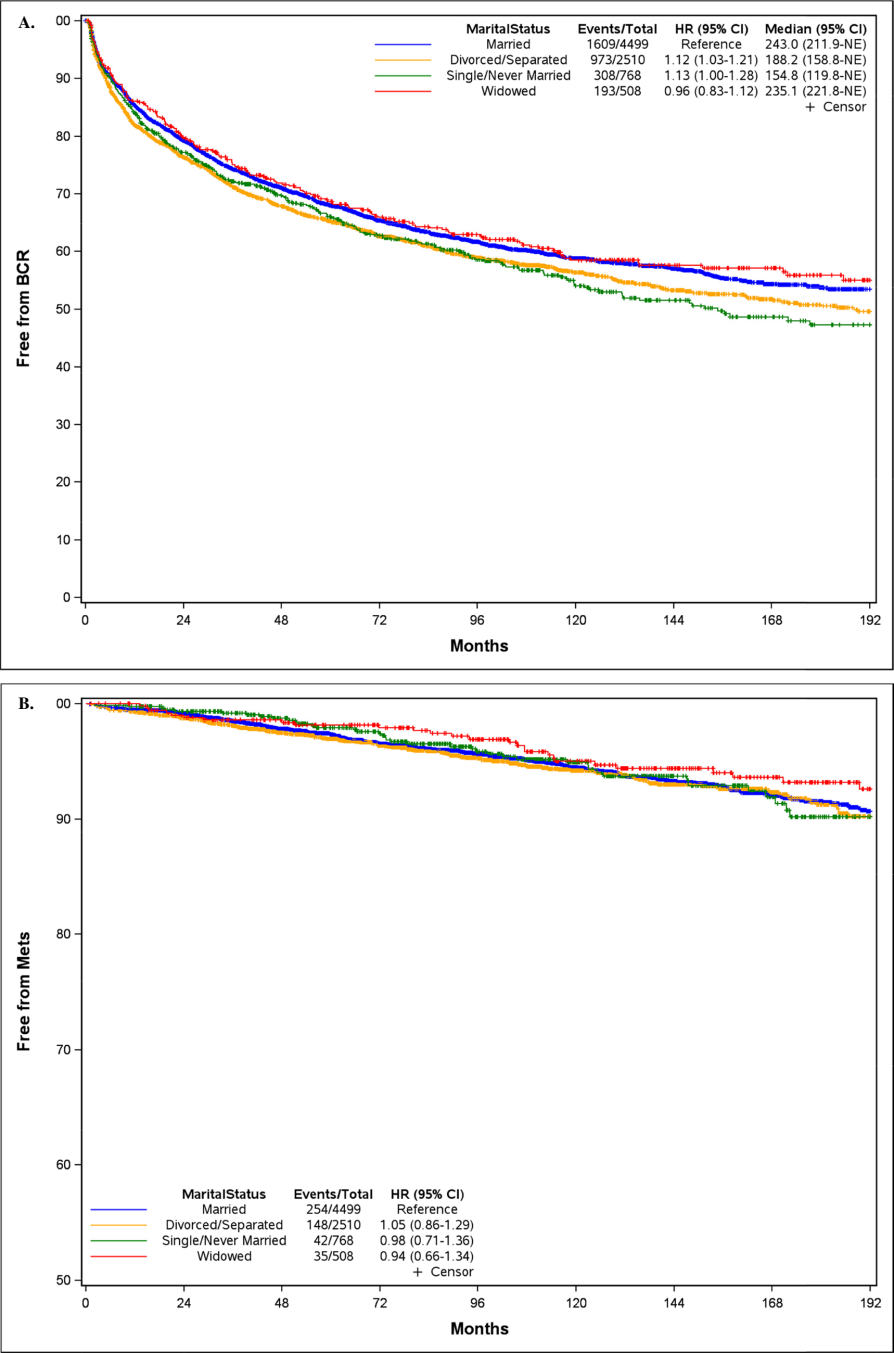

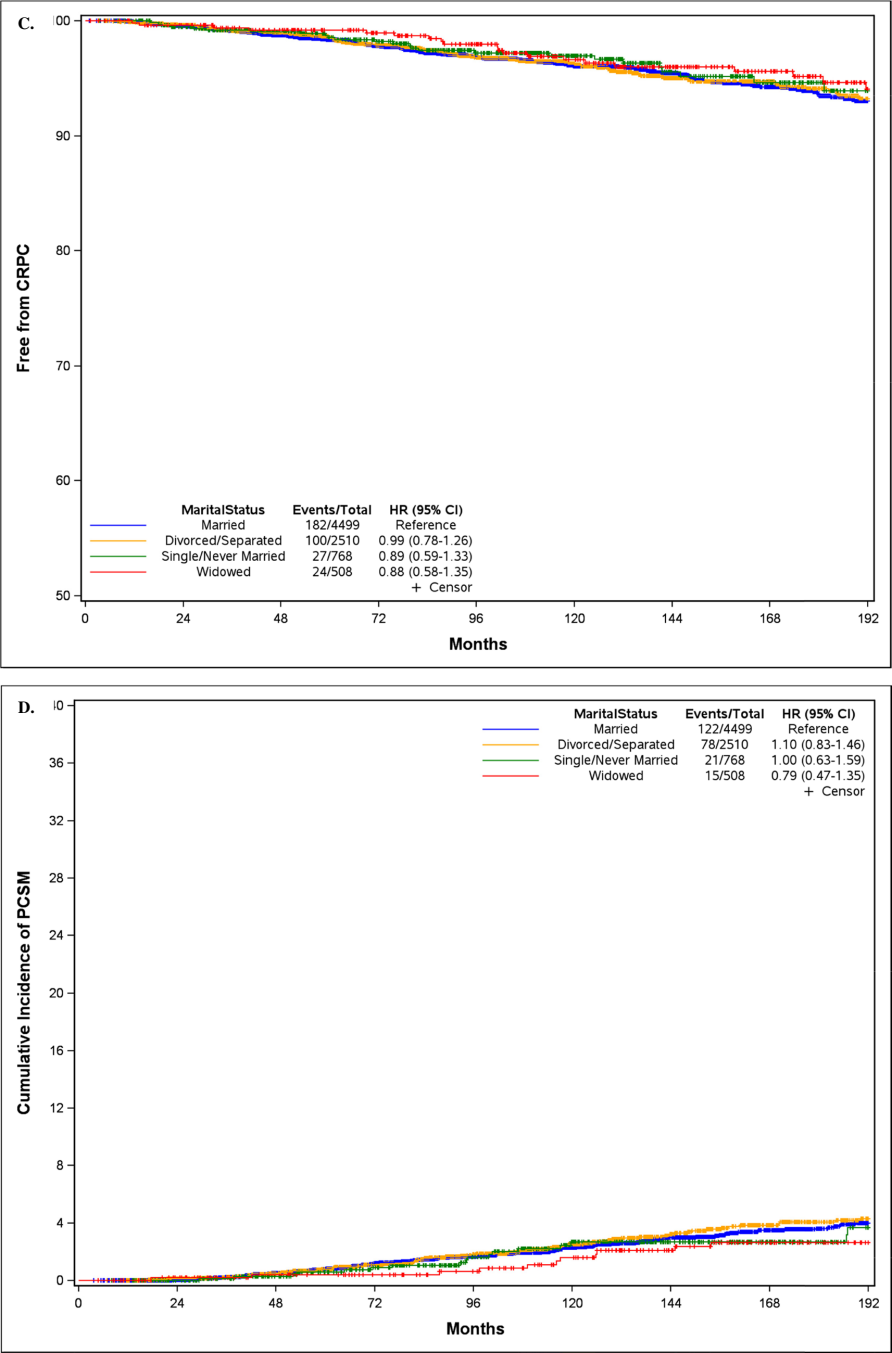
Kaplan–Meier curves stratified by marital status for time to (**A**) biochemical recurrence, (**B**) metastasis, (**C**) castration-resistant prostate cancer, and (**D**) prostate cancer specific mortality

As evident in Fig. 2A, the median time to BCR was significantly shorter for divorced/separated men (188.2 months) and single/never married men (154.8 months) compared to married men (243.0 months). Consistent with these findings were those from univariable Cox analyses: compared to married men, divorced/separated men had higher risk of BCR (HR = 1.12; 95% CI 1.03–1.21), as did single/never married men (HR = 1.13; 95% CI 1.00–1.28). The median time to BCR did not differ significantly between widowers (235.1 months) and married men. In contrast, there were no overall significant associations between marital status and time to metastasis, CRPC, or PCSM, nor were any individual marital status groups associated with these later outcomes (all p > 0.05) (Table 2).

**Table 2:** Univariable and multivariable associations between marital status and risk of BCR, Mets, CRPC, and PCSM

			**Univariable**		Multivariable*		Multivariable**	
**Outcomes**	**Marital Status**	**Events (%)**	**HR (95% CI)**	**p-value**	**HR (95% CI)**	**p-value**	**HR (95% CI)**	**p-value**
BCR	Married	1,609 (36%)	Ref	0.014	Ref	0.329	Ref	0.613
	Divorced/Separated	973 (39%)	1.12 (1.03, 1.21)		1.04 (0.96, 1.13)		1.05 (0.97, 1.13)	
	Single/Never Married	308 (40%)	1.13 (1.00, 1.28)		1.06 (0.94, 1.20)		1.00 (0.89, 1.14)	
	Widowed	193 (38%)	0.96 (0.83, 1.12)		0.92 (0.79, 1.06)		0.96 (0.83, 1.12)	
Mets	Married	254 (6%)	Ref	0.927	Ref	0.960	Ref	0.826
	Divorced/Separated	148 (6%)	1.05 (0.86, 1.29)		0.98 (0.80, 1.20)		1.07 (0.87, 1.31)	
	Single/Never Married	42 (6%)	0.98 (0.71, 1.36)		0.97 (0.70, 1.34)		0.91 (0.66, 1.26)	
	Widowed	35 (7%)	0.94 (0.66, 1.34)		0.91 (0.63, 1.30)		1.02 (0.72, 1.46)	
CRPC	Married	182 (4%)	Ref	0.892	Ref	0.632	Ref	0.724
	Divorced/Separated	100 (4%)	0.99 (0.78, 1.26)		0.88 (0.69, 1.12)		0.98 (0.77, 1.25)	
	Single/Never Married	27 (4%)	0.89 (0.59, 1.33)		0.87 (0.58, 1.31)		0.79 (0.53, 1.18)	
	Widowed	24 (5%)	0.88 (0.58, 1.35)		0.82 (0.54, 1.27)		0.96 (0.63, 1.47)	
PCSM	Married	122 (3%)	Ref	0.683	Ref	0.639	Ref	0.722
	Divorced/Separated	78 (3%)	1.10 (0.83, 1.46)		1.01 (0.76, 1.36)		1.12 (0.83, 1.50)	
	Single/Never Married	21 (3%)	1.00 (0.63, 1.59)		1.05 (0.66, 1.66)		0.90 (0.57, 1.45)	
	Widowed	15 (3%)	0.79 (0.47, 1.35)		0.71 (0.41, 1.23)		0.87 (0.51, 1.50)	

^*^Adjusted for age, race, year of surgery, biopsy Gleason score, clinical stage, and log-transformed PSA

^**^Adjusted for RP Gleason score, log-transformed PSA, seminal vesicle invasion, positive surgical margin, lymph node involvement, extracapsular extension, and pathological stage

On multivariable analyses, after adjusting for clinical characteristics and further adjusting for pathological features, the significant associations with BCR were lost and no longer significant (all p > 0.05) (Table 2). Likewise, there remained no significant associations between marital status as a whole or any specific groups and late outcomes including metastasis, CRPC, or PCSM. Results were essentially identical after adjusting for the time from diagnosis to RP (Table 3).

**Table 3:** Univariable and multivariable associations between marital status and risk of BCR, Mets, CRPC, and PCSM additionally adjusted for time from PC diagnosis to RP among a subset of patients (N = 8218) who had time from diagnosis to RP

			**Univariable**		Multivariable*		Multivariable**	
**Outcomes**	**Marital Status**	**Events (%)**	**HR (95% CI)**	**p-value**	**HR (95% CI)**	**p-value**	**HR (95% CI)**	**p-value**
BCR	Married	1,599 (36%)	Ref	0.023	Ref	0.374	Ref	0.576
	Divorced/Separated	960 (39%)	1.10 (1.02, 1.20)		1.04 (0.96, 1.13)		1.05 (0.97, 1.14)	
	Single/Never Married	304 (40%)	1.13 (1.00, 1.28)		1.06 (0.94, 1.20)		1.01 (0.89, 1.14)	
	Widowed	193 (38%)	0.97 (0.83, 1.13)		0.92 (0.79, 1.07)		0.96 (0.83, 1.11)	
Mets	Married	253 (6%)	Ref	0.949	Ref	0.958	Ref	0.868
	Divorced/Separated	146 (6%)	1.04 (0.85, 1.28)		0.97 (0.79, 1.19)		1.06 (0.86, 1.30)	
	Single/Never Married	42 (6%)	0.98 (0.71, 1.36)		0.97 (0.70, 1.35)		0.91 (0.66, 1.27)	
	Widowed	35 (7%)	0.94 (0.66, 1.34)		0.91 (0.64, 1.31)		1.01 (0.71, 1.44)	
CRPC	Married	181 (4%)	Ref	0.913	Ref	0.581	Ref	0.735
	Divorced/Separated	98 (4%)	0.98 (0.77, 1.25)		0.86 (0.67, 1.10)		0.96 (0.75, 1.23)	
	Single/Never Married	27 (4%)	0.90 (0.60, 1.34)		0.87 (0.58, 1.31)		0.79 (0.53, 1.19)	
	Widowed	24 (5%)	0.89 (0.58, 1.36)		0.83 (0.54, 1.28)		0.95 (0.62, 1.46)	
PCSM	Married	121 (3%)	Ref	0.702	Ref	0.670	Ref	0.692
	Divorced/Separated	77 (3%)	1.10 (0.83, 1.47)		1.01 (0.75, 1.36)		1.13 (0.84, 1.52)	
	Single/Never Married	21 (3%)	1.01 (0.64, 1.61)		1.05 (0.66, 1.67)		0.92 (0.57, 1.47)	
	Widowed	15 (3%)	0.80 (0.47, 1.36)		0.72 (0.42, 1.25)		0.86 (0.50, 1.48)	

^*^Adjusted for age, race, year of surgery, biopsy Gleason score, clinical stage, log-transformed PSA, and log-transformed days from diagnosis to RP

^**^Adjusted for RP Gleason score, log-transformed PSA, seminal vesicle invasion, positive surgical margin, lymph node involvement, extracapsular extension, pathological stage, and log-transformed days from diagnosis to RP

## Discussion

In this study, we tested if there was an association between marital status and post-RP outcomes in an equal-access cohort of patients from the VAHCS. Marital status/support has been shown to play an important role in cancer outcomes due to the instrumental support a spouse can provide that helps mitigate the stress that comes with the diagnosis and treatment of cancer[24]. Univariable analysis in this study showed that marital status was significantly associated with BCR among patients with localized prostate cancer undergoing RP with single and divorced patients having higher risks, but no other associations were observed with later outcomes. Importantly, when adjusting for baseline covariates, these results were no longer significant. These findings suggest that marital status does not affect post RP outcomes in this Veteran cohort.

Similar to this study’s results, Schiffman et al. reported no significant impact of marital status on biochemical recurrence, metastases, or death after RP in a cohort of males from a high-volume center. In addition, the authors found that marital status was unrelated to clinical and pathological characteristics in patients[25]. Khan et al. also examined the association between marital status, all-cause mortality and PCSM [26]. They found that men who were unmarried were at an increased risk for all-cause mortality and that unmarried status was associated with PCSM compared to married. It is important to note that authors Schiffman and Khan used data from one institution, whereas this study used data from nine VA centers[27].

Researchers who found marital status influences cancer-related outcomes due to social support suggest that unmarried men could benefit from cancer prevention and management interventions to improve outcomes[12]. The healthcare system plays a significant role in enhancing cancer screenings, diagnosis, and the quality of clinical care received. A report investigating cancer outcomes by medical insurance type found that the VA excelled in diagnosing cancer early and that VA patients were more likely to receive treatment according to current cancer guidelines compared to patients with other insurance types[28, 29]. However, the report also found that VA patients had the longest time for treatment compared to different groups. Overall, this report found that patients with VA insurance experienced comparable, if not better, disease prognosis and treatment outcomes compared to patients with other insurance types, which speaks to the improved efficient and equitable quality of care received from the VA Health Care System VAHCS[30].

Equal healthcare systems such as the VA establish evidence-based processes that ensure each patient receives the same high-quality care[31]. The resources and support provided by an equal access health care system can potentially supplement the support a patient with a spouse or significant other may receive[32]. Having resources in place that can help patients with transportation, paperwork, and in-person visits can benefit patients and help them solely focus on their treatment and getting better[33]. In addition, equal access helps with disparities seen in treatments, which have been shown to significantly impact survival rates and quality of life for affected individuals[34–36]. For example, Guerrios-Rivera et al. found no evidence of worse outcomes for Hispanic men compared to non-White Hispanic men undergoing RP from multiple equal access healthcare systems[22]. Additional studies have also confirmed that Black men who receive care from an equal access clinic may have similar or better outcomes to systemic therapies when compared to White men[37]. These results highlight the importance of prioritizing equal access to healthcare for everyone, as it can lead to improved health outcomes across the board.

Baseline comparisons revealed shorter times from diagnosis to RP among Widowers and longer times to RP among those who were divorced/separated. While outside of the scope of the current manuscript, an interesting avenue for future work would be to investigate the association between marital status and time from diagnosis to treatment among patients with PC.

One limitation of our study is that we were unable to capture additional support outside of marriage that patients may have received. For instance, patients who reported being single may have been living with a romantic partner, friend, or family member(s) from which they received support. Moreover, patients who were married were likely to have varied levels of support not captured. Importantly, our study examined marital status—a demographic indicator of self-reported relationship category—and not marital satisfaction, perceived partner support, or relationship quality, which are distinct constructs that would require dedicated measures and study designs. Our null findings should therefore be interpreted as evidence that marital status, as a structural variable, is not associated with post-RP outcomes in this VA cohort; they should not be interpreted as evidence about whether the quality of a patient’s relationship or the degree of partner support affects outcomes. Examining marital satisfaction and perceived support as predictors of post-RP outcomes is an important direction for future research. Other limitations of this study included the inability to assess the type and extent of additional social support received by patients from the VA fully. The VAHCS offers support services such as telecommunication, delivering home care to the patient, financial assistance, and emotional and practical support provided by oncology social workers who offer counseling, support groups, and resource navigation[38]. In addition, our primary focus was on men undergoing RP, which may not be representative of patients receiving other forms of therapy. We did not gather information regarding non-spousal support available to patients, such as family or friends. This information could have provided insight into the social support of patients who were not married. This gap in data limits our ability to draw a definitive conclusion about the overall impact of social support on RP outcomes. Still, it provides an area of opportunity for future studies. Despite these limitations, the study's strengths included data from patients who had equal access to healthcare.

We found no association between marital status as a structural demographic variable and post-RP outcomes in patients with localized prostate cancer in the VAHCS. These findings should not be extrapolated to relationship quality or perceived partner support, which were not assessed in this study. Whether marital satisfaction, perceived partner support, or other dimensions of social support affect post-RP outcomes remains an open question, and future studies using validated instruments for these constructs are warranted. Whether our null finding reflects a true absence of association between marital status and RP outcomes in general, or is influenced by the supportive services available within the VAHCS, also remains to be determined.

## Data Availability

These data were obtained from a prospectively collected database from multiple Veterans Affairs health systems. Data are available on request within Veterans Affairs rules and policy requirements.
